# 2-Cyano­anilinium iodide

**DOI:** 10.1107/S1600536813019314

**Published:** 2013-07-20

**Authors:** David J. Vumbaco, Michael N. Kammer, Lynn V. Koplitz, Joel T. Mague

**Affiliations:** a Department of Biological Sciences, Loyola University, New Orleans, LA 70118, USA; b Department of Physics, Loyola University, New Orleans, LA 70118, USA; c Department of Chemistry, Loyola University, New Orleans, LA 70118, USA; d Department of Chemistry, Tulane University, New Orleans, LA 70118, USA

## Abstract

The solid-state structure of the title salt, C_7_H_7_N_2_^+.^I^−^, consists of cation–anion sheets lying parallel to (110), with the components linked by N—H⋯I hydrogen bonds.

## Related literature

For the structure of 2-cyano-1-methyl­pyridinium iodide, see: Kammer *et al.* (2013 ▶). For structures of other 2-cyano­anilinium salts, see: Cui & Chen (2010 ▶); Zhang (2009 ▶); Cui & Wen (2008 ▶); Oueslati *et al.* (2005 ▶). For the structures of 4-cyanoanilinium halides, see: Mague *et al.* (2012 ▶); Vumbaco *et al.* (2012 ▶); Colapietro *et al.* (1981 ▶).
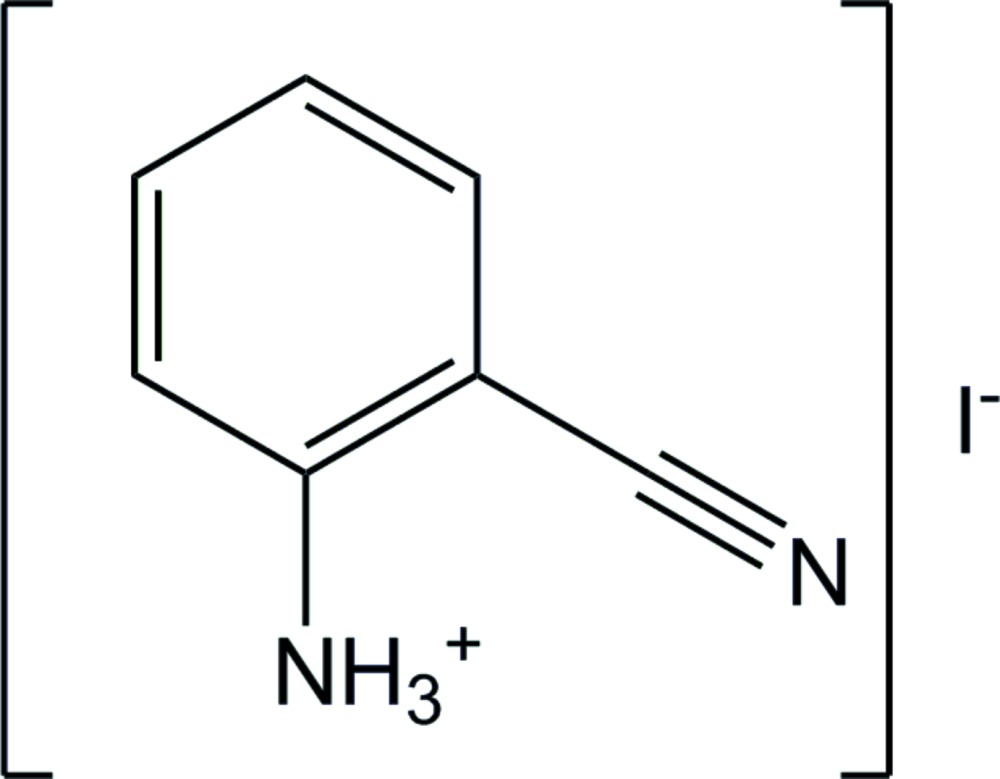


## Experimental

### 

#### Crystal data

C_7_H_7_N_2_^+^·I^−^
*M* *_r_* = 246.05
Orthorhombic, 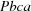

*a* = 10.1474 (15) Å
*b* = 8.6979 (13) Å
*c* = 18.073 (3) Å
*V* = 1595.2 (4) Å^3^
*Z* = 8
Mo *K*α radiation
μ = 3.94 mm^−1^
*T* = 100 K
0.20 × 0.19 × 0.16 mm

#### Data collection

Bruker SMART APEX CCD diffractometer
Absorption correction: numerical (*SADABS*; Bruker, 2010 ▶) *T*_min_ = 0.43, *T*_max_ = 0.58
25911 measured reflections
2112 independent reflections
2030 reflections with *I* > 2σ(*I*)
*R*_int_ = 0.039

#### Refinement

*R*[*F* ^2^ > 2σ(*F* ^2^)] = 0.016
*wR*(*F* ^2^) = 0.041
*S* = 1.10
2112 reflections
92 parameters
H-atom parameters constrained
Δρ_max_ = 0.59 e Å^−3^
Δρ_min_ = −0.58 e Å^−3^

### 

Data collection: *APEX2* (Bruker, 2010 ▶); cell refinement: *SAINT* (Bruker, 2010 ▶); data reduction: *SAINT*; program(s) used to solve structure: *SHELXM* (Sheldrick, 2008 ▶); program(s) used to refine structure: *SHELXL2013* (Sheldrick, 2008 ▶); molecular graphics: *DIAMOND* (Brandenburg & Putz, 2012 ▶); software used to prepare material for publication: *SHELXTL* (Sheldrick, 2008 ▶).

## Supplementary Material

Crystal structure: contains datablock(s) global, I. DOI: 10.1107/S1600536813019314/hb7106sup1.cif

Structure factors: contains datablock(s) I. DOI: 10.1107/S1600536813019314/hb7106Isup2.hkl

Supplementary material file. DOI: 10.1107/S1600536813019314/hb7106Isup3.cml

Additional supplementary materials:  crystallographic information; 3D view; checkCIF report

## Figures and Tables

**Table 1:** Hydrogen-bond geometry (Å, °)

*D*—H⋯*A*	*D*—H	H⋯*A*	*D*⋯*A*	*D*—H⋯*A*
N1—H1*A*⋯I1	0.88	2.74	3.6069 (13)	169
N1—H1*B*⋯I1^i^	0.88	2.71	3.5501 (14)	160
N1—H1*C*⋯I1^ii^	0.88	2.84	3.6615 (13)	156

Symmetry codes: (i) 
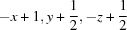
; (ii) 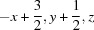.

**Fig. 1.**
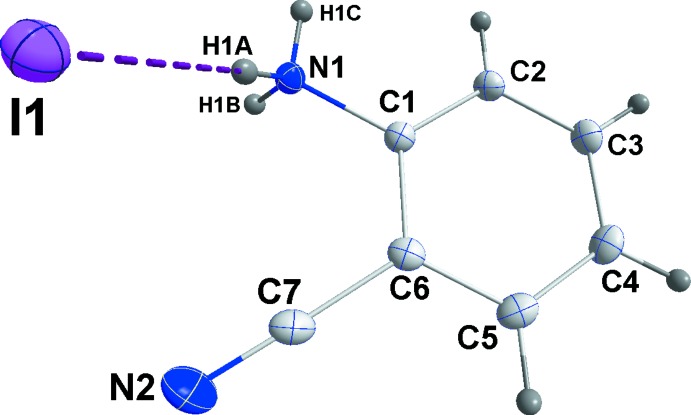
Perspective view of the cation–anion pair showing the shortest interionic N—H···I hydrogen bonds and 50% probability displacement ellipsoids.

**Fig. 2.**
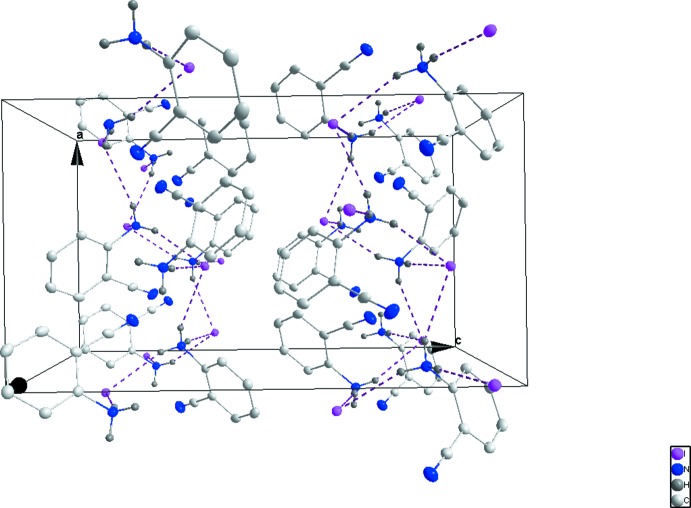
Packing viewed down the b axis with N—H···I interactions shown as dotted lines.

**Fig. 3.**
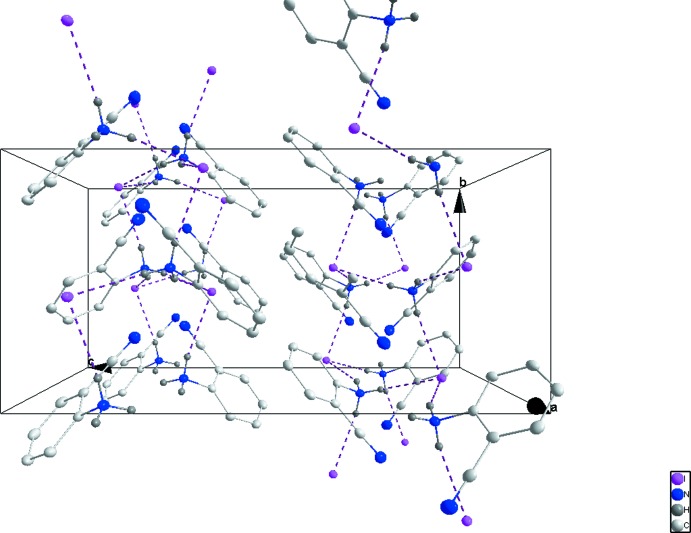
Packing viewed down the a axis with N—H···I interactions shown as dotted lines.

## supplementary crystallographic information

### Comment

In the solid state, salts of the isomeric cyanoanilinium ions exhibit both layer and network structures. Thus, the iodide (Mague *et al.*, 2012) and chloride (Colapietro *et al.*, 1981) salts of the 4-cyanoanilinium ion as well as the chloride (Oueslati, *et al.*, 2005) and nitrate (Cui & Wen, 2008) salts of the 2-cyanoanilinium ion have layer structures in which the –NH_3_^+^ and anion moieties form a double layer with the organic portion of the cations protruding perpendicularly from both sides of this double layer. By contrast, the bromide (Zhang, 2009) and perchlorate (Cui & Chen, 2010) salts of the 2-cyanoanilinium ion form network structures while 4-cyanoanilinium bromide (Vumbaco *et al.*, 2012) forms a stepped layer structure. A different structure type is found in the title compound where the basic unit is a zigzag chain of alternating cations and anions running parallel to *a* assembled by alternating short and long N—H···I hydrogen bonds (Table 1, Fig. 1). These chains are assembled into sheets parallel to (110) by intermediate length N—H···I hydrogen bonds between cations in one chain and anions in the next in which the cations in each chain are arranged in an "umbrella" fashion (Fig. 3) instead of projecting straight out towards the edges of the layer. It is also significantly different from the structure adopted by the isomeric compound 2-cyano-*N*-methylpyridinium iodide (Kammer *et al.*, 2013), at least in part because the intermolecular C—H···I interactions in this compound are expected to be weaker than the N—H···I interactions in the title compound.

### Experimental

2-Cyanoaniline (0.55 g) and 1.0 ml of aqueous hydroiodic acid (47% by mass) were combined in 10 ml of ethanol. This solution was slowly evaporated to dryness at room temperature to form colourless blocks of the title compound.

### Refinement

H-atoms attached to carbon were placed in calculated positions (C—H = 0.95 Å) while those attached to nitrogen were placed in locations derived from a difference map and their coordinates then adjusted to give N—H = 0.88 Å. All were included as riding contributions with isotropic displacement parameters 1.2 times those of the attached atoms.

### Crystal data

**Table:**

C_7_H_7_N_2_^+^·I^−^	*D*_x_ = 2.049 Mg m^−^^3^
*M**_r_* = 246.05	Mo *K*α radiation, λ = 0.71073 Å
Orthorhombic, *P**b**c**a*	Cell parameters from 9853 reflections
*a* = 10.1474 (15) Å	θ = 2.3–29.1°
*b* = 8.6979 (13) Å	µ = 3.94 mm^−^^1^
*c* = 18.073 (3) Å	*T* = 100 K
*V* = 1595.2 (4) Å^3^	Block, colourless
*Z* = 8	0.20 × 0.19 × 0.16 mm
*F*(000) = 928	

### Data collection

**Table:**

Bruker SMART APEX CCD diffractometer	2112 independent reflections
Radiation source: fine-focus sealed tube	2030 reflections with *I* > 2σ(*I*)
Graphite monochromator	*R*_int_ = 0.039
Detector resolution: 8.3660 pixels mm^-1^	θ_max_ = 29.1°, θ_min_ = 2.3°
φ and ω scans	*h* = −13→13
Absorption correction: numerical (*SADABS*; Bruker, 2010)	*k* = −11→11
*T*_min_ = 0.43, *T*_max_ = 0.58	*l* = −24→24
25911 measured reflections	

### Refinement

**Table:**

Refinement on *F*^2^	Secondary atom site location: difference Fourier map
Least-squares matrix: full	Hydrogen site location: mixed
*R*[*F*^2^ > 2σ(*F*^2^)] = 0.016	H-atom parameters constrained
*wR*(*F*^2^) = 0.041	*w* = 1/[σ^2^(*F*_o_^2^) + (0.0208*P*)^2^ + 0.7585*P*] where *P* = (*F*_o_^2^ + 2*F*_c_^2^)/3
*S* = 1.10	(Δ/σ)_max_ = 0.002
2112 reflections	Δρ_max_ = 0.59 e Å^−^^3^
92 parameters	Δρ_min_ = −0.58 e Å^−^^3^
0 restraints	Extinction correction: *SHELXL2013* (Sheldrick, 2008), Fc^*^=kFc[1+0.001xFc^2^λ^3^/sin(2θ)]^-1/4^
Primary atom site location: structure-invariant direct methods	Extinction coefficient: 0.00151 (13)

### Special details

**Table:**

Experimental. The diffraction data were obtained from 3 sets of 400 frames, each of width 0.5° in ω, colllected at φ = 0.00, 90.00 and 180.00° and 2 sets of 800 frames, each of width 0.45° in φ, collected at ω = -30.00 and 210.00°. The scan time was 10 sec/frame.
Geometry. All e.s.d.'s (except the e.s.d. in the dihedral angle between two l.s. planes) are estimated using the full covariance matrix. The cell e.s.d.'s are taken into account individually in the estimation of e.s.d.'s in distances, angles and torsion angles; correlations between e.s.d.'s in cell parameters are only used when they are defined by crystal symmetry. An approximate (isotropic) treatment of cell e.s.d.'s is used for estimating e.s.d.'s involving l.s. planes.

### Fractional atomic coordinates and isotropic or equivalent isotropic displacement parameters (Å^2^)

**Table:**

	*x*	*y*	*z*	*U*_iso_*/*U*_eq_	
I1	0.58823 (2)	0.05477 (2)	0.13679 (2)	0.01326 (5)	
N1	0.58054 (12)	0.44877 (14)	0.19889 (8)	0.0140 (3)	
H1A	0.5870	0.3496	0.1900	0.017*	
H1B	0.5590	0.4716	0.2448	0.017*	
H1C	0.6593	0.4909	0.1973	0.017*	
N2	0.28338 (14)	0.23930 (17)	0.21818 (8)	0.0246 (3)	
C1	0.49549 (15)	0.51204 (18)	0.14089 (7)	0.0129 (3)	
C2	0.54089 (14)	0.63213 (16)	0.09804 (8)	0.0149 (3)	
H2	0.6252	0.6758	0.1071	0.018*	
C3	0.46120 (15)	0.68861 (17)	0.04120 (8)	0.0170 (3)	
H3	0.4916	0.7711	0.0113	0.020*	
C4	0.33787 (15)	0.62516 (17)	0.02809 (8)	0.0183 (3)	
H4	0.2854	0.6627	−0.0115	0.022*	
C5	0.29073 (15)	0.50692 (19)	0.07257 (8)	0.0172 (3)	
H5	0.2054	0.4654	0.0643	0.021*	
C6	0.36974 (17)	0.44965 (16)	0.12950 (8)	0.0141 (3)	
C7	0.32073 (14)	0.33165 (17)	0.17807 (8)	0.0167 (3)	

### Atomic displacement parameters (Å^2^)

**Table:**

	*U*^11^	*U*^22^	*U*^33^	*U*^12^	*U*^13^	*U*^23^
I1	0.01359 (7)	0.01201 (7)	0.01419 (7)	−0.00040 (3)	−0.00112 (3)	−0.00004 (3)
N1	0.0147 (6)	0.0120 (6)	0.0152 (6)	−0.0004 (4)	−0.0008 (4)	0.0005 (4)
N2	0.0270 (7)	0.0236 (7)	0.0233 (7)	−0.0071 (6)	0.0076 (6)	−0.0023 (6)
C1	0.0145 (7)	0.0117 (7)	0.0126 (6)	0.0012 (5)	0.0003 (5)	−0.0022 (5)
C2	0.0153 (6)	0.0120 (6)	0.0174 (6)	−0.0003 (5)	0.0003 (5)	−0.0002 (5)
C3	0.0210 (7)	0.0127 (7)	0.0174 (7)	0.0019 (6)	0.0010 (5)	0.0009 (5)
C4	0.0213 (7)	0.0163 (7)	0.0174 (6)	0.0050 (6)	−0.0035 (5)	−0.0020 (6)
C5	0.0150 (6)	0.0166 (7)	0.0199 (7)	0.0020 (6)	−0.0011 (5)	−0.0058 (6)
C6	0.0142 (8)	0.0119 (7)	0.0161 (7)	0.0012 (5)	0.0029 (5)	−0.0033 (5)
C7	0.0143 (6)	0.0174 (7)	0.0182 (7)	−0.0017 (5)	0.0023 (5)	−0.0053 (6)

### Geometric parameters (Å, º)

**Table:**

N1—C1	1.4651 (19)	C2—H2	0.9500
N1—H1A	0.8800	C3—C4	1.388 (2)
N1—H1B	0.8800	C3—H3	0.9500
N1—H1C	0.8800	C4—C5	1.390 (2)
N2—C7	1.146 (2)	C4—H4	0.9500
C1—C2	1.380 (2)	C5—C6	1.396 (2)
C1—C6	1.402 (2)	C5—H5	0.9500
C2—C3	1.397 (2)	C6—C7	1.439 (2)
			
C1—N1—H1A	106.4	C4—C3—H3	119.7
C1—N1—H1B	116.3	C2—C3—H3	119.7
H1A—N1—H1B	114.3	C3—C4—C5	120.37 (14)
C1—N1—H1C	110.8	C3—C4—H4	119.8
H1A—N1—H1C	109.6	C5—C4—H4	119.8
H1B—N1—H1C	99.3	C4—C5—C6	119.51 (14)
C2—C1—C6	120.98 (13)	C4—C5—H5	120.2
C2—C1—N1	119.30 (13)	C6—C5—H5	120.2
C6—C1—N1	119.72 (13)	C5—C6—C1	119.52 (14)
C1—C2—C3	119.07 (14)	C5—C6—C7	120.37 (15)
C1—C2—H2	120.5	C1—C6—C7	120.07 (14)
C3—C2—H2	120.5	N2—C7—C6	178.30 (17)
C4—C3—C2	120.51 (14)		
			
C6—C1—C2—C3	−1.9 (2)	C4—C5—C6—C7	177.45 (14)
N1—C1—C2—C3	177.87 (13)	C2—C1—C6—C5	1.9 (2)
C1—C2—C3—C4	0.2 (2)	N1—C1—C6—C5	−177.89 (13)
C2—C3—C4—C5	1.6 (2)	C2—C1—C6—C7	−175.70 (13)
C3—C4—C5—C6	−1.6 (2)	N1—C1—C6—C7	4.5 (2)
C4—C5—C6—C1	−0.1 (2)		

### Hydrogen-bond geometry (Å, º)

**Table:**

*D*—H···*A*	*D*—H	H···*A*	*D*···*A*	*D*—H···*A*
N1—H1*A*···I1	0.88	2.74	3.6069 (13)	169
N1—H1*B*···I1^i^	0.88	2.71	3.5501 (14)	160
N1—H1*C*···I1^ii^	0.88	2.84	3.6615 (13)	156

Symmetry codes: (i) −*x*+1, *y*+1/2, −*z*+1/2; (ii) −*x*+3/2, *y*+1/2, *z*.
